# The association between RCAS1 expression in laryngeal and pharyngeal cancer and its healthy stroma with cancer relapse

**DOI:** 10.1186/1471-2407-9-35

**Published:** 2009-01-28

**Authors:** Magdalena Dutsch-Wicherek, Romana Tomaszewska, Agata Lazar, Lukasz Wicherek, Jacek Skladzien

**Affiliations:** 1 ENT Head and Neck Surgery Department, the Jagiellonian University, Krakow, Poland; 2 Pathomorphology Department, the Jagiellonian University, Krakow, Poland; 3 Gynecology Oncology Department, the Jagiellonian University, Krakow, Poland

## Abstract

**Background:**

The purpose of this study has been to establish the level of RCAS1 – a membrane protein expressed in various cancer cells and able to induce apoptosis of CTLs and NK cells in pharyngeal and laryngeal cancer and its clear surgical margin – with respect to clinicopathological features and to patient's follow up and evaluate its possible role in cancer relapse.

**Methods:**

A total of 122 tissue samples were obtained: 51 samples from laryngeal and pharyngeal squamous cell carcinoma, 51 samples from the clear surgical margins of these tumors, and 20 tissue samples derived from the healthy mucous membranes of the upper respiratory tract mucosa of patients without cancerous tumors. Patients were observed for a total of 4 years following surgical treatment. The level of RCAS1 expression was assessed by immunohistochemistry and Western blot.

**Results:**

RCAS1 was identified in all laryngeal and pharyngeal carcinomas and in almost all the clear surgical margin samples. The level of RCAS1 expression was significantly higher in the cancerous samples than in the clear surgical margins and was determined to be related to the grade of the cancer and the presence of lymph node metastases. In cases of cancer relapse, significantly higher levels of RCAS1 expression were observed in the clear surgical margins.

**Conclusion:**

Selective cytotoxic immune cell suppression concomitant with tumor growth and associated with RCAS1 expression seems to be an important event connected with cancer relapse.

## Background

Head and neck squamous cell carcinoma constitutes the sixth most common type of cancer in the world [1]. The five-year survival rate for patients with this disease has not improved appreciably since the early 1980s, remaining at approximately 52% [2,3]. A common problem in cases of head and neck cancer is the recurrence of the disease either in the location of primary surgical resection (although the surgical resection margins were histopathologically free of cancer cells) or in another place. The risk of a local recurrence of the disease is still high, approximately 10–30% [4].

Tumor-associated antigen RCAS1 (receptor-binding cancer antigen expressed on SiSo cells) is a type-II membrane protein, expressed in various human cancer cells [5]. It has also been detected in blood serum in a soluble form (sRCAS1) that is probably released from the membrane form as a result of ectodomain shedding [6,7]. The RCAS1 protein acts as a ligand for a putative receptor present on various human cells, including normal peripheral lymphocytes, such as T, B, and NK (natural killer) cells. RCAS1 inhibits the growth of receptor-expressing cells both *in vitro *and *in vivo *and induces apoptotic cell death through the activation of caspase 3 and the collapse of mitochondrial transmembrane potential [8,9]. RCAS1 is reportedly expressed with high frequency in breast, lung, laryngeal, pharyngeal, gallbladder, ovarian, uterine, and bile duct cancer cells as well as in Reed Sternberg cells and erythroid progenitor cells [8-18].

RCAS1 expression has also reportedly been associated with the markers of poor prognosis in various types of cancers, such as the presence of lymph node metastases in gallbladder cancer [12] and the high grade of tumor cells in ductal breast and lung cancers [19,10]; it has additionally been associated with the presence of tumor invasion in vessels and nerves, and with the depth of tumor invasion in gallbladder cancer [12]. Moreover, RCAS1 expression has been identified as an independent, negative prognostic factor in both gallbladder and esophageal cancers [12,20]. Furthermore, the RCAS1 blood serum level has been linked with tumor progression in endometrial cancer [7]. In our recent study, the soluble form of RCAS1 was detected in the blood serum of patients with head and neck cancers, while an increase in the sRCAS1 level was connected with cancer relapse [21]. Sonoda *et al. *has pointed out that RCAS1 expression may facilitate cancer cell invasion of connective tissue through the enhancement of tumor invasive potency by inducing stromal tissue remodeling and the evasion of antitumor immune surveillance [6].

As RCAS1 expression has not been analyzed in pharyngeal and laryngeal squamous cell carcinomas and their healthy stroma, yet has been shown to be involved in tumor invasion, we decided that the levels of RCAS1 expression should be analyzed with respect to the clinicopathological features and that patients who underwent surgery due to pharyngeal and laryngeal squamous cell carcinomas should be followed up for 4 years.

## Methods

### Clinical material

All patients in this study with head and neck squamous cell carcinoma, including pharyngeal (comprises the mesopharynx and hypopharynx), and laryngeal cancers, underwent surgery between September 2002 and November 2003 at the Department of Otolaryngology and Head and Neck Surgery of the Jagiellonian University in Krakow. The patient's informed consent was obtained in each case. Additionally, approval for the research program was obtained from the Ethical Committee of the Jagiellonian University in Krakow: KBET/379/13/2003. Patients were randomly selected and observed for a total of 4 years following surgical treatment. The observations were completed on the 15^th ^of December 2007. A total of 122 tissue samples were obtained: 51 samples from laryngeal and pharyngeal squamous cell carcinoma, 51 samples from the clear surgical margins of these tumors, and 20 tissue samples derived from the healthy mucous membranes of the upper respiratory tract mucosa of patients without cancerous lesions (these patients had been operated on due to nasal septum deviation). The surgically removed material was evaluated in order to determine the histological type and metastases of the lymph nodes using histological methods in the Pathology Department of the Jagiellonian University. Directly following the surgical procedure, the pathologist collected the cancer tissue samples and their clear surgical margin samples for examination. From each patient with laryngeal and pharyngeal cancer, two samples were collected, one derived from the cancerous tissue and the other from its clear surgical margin. The clear surgical margin was defined as the 1 cm^2 ^area of tumor-adjacent tissue macroscopically and histologically free of any neoplasmatic texture. The line of surgical resection was also macroscopically and histologically free of cancerous texture.

### The group of patients

The characteristics of the patient groups are demonstrated in detail in Table 1. In each case, squamous cell carcinoma was identified. The cases were staged in accordance with the TNM Classification of Malignant Tumors, 5th edition (1997). All patients underwent primary surgical treatment, and 28 received radiation therapy following the operation because of lymph node metastases. The survival time was defined as the time from the date of surgery to the final date of observation in this study (the 15^th ^of December, 2007).

**Table 1:** Clinicopathological variables for patients with laryngeal and pharyngeal cancer.

Clinicopathologic variable	No. of patients
Age (years)	55.3 (42–74)
Men	59.6
Women	53.8

Sex	
Men	41
Women	10

Lymph node metastasis	
Negative	24
Positive	27

Positive lymph node metastasis without extracapsular spread	11
Positive lymph node metastases with extracapsular spread	16

Tumor size	
T1	2
T2	22
T3	23
T4	4

Tumor grade	
1	1
2	31
3	19

Pharyngeal cancer	21
Laryngeal cancer	23
Cancer of pharynx and larynx	7

Type of surgery	
Total laryngectomies	16
Partial laryngectomies	7
Partial pharyngectomies	21
Laryngopharyngectomies	7
Radiation therapy following surgery	28

### Preparation of tissue extracts and Western blotting

The Western blot analysis was performed in the Department of Analytical Biochemistry, ENT Head and Neck Surgery Department, and the Gynecology and Infertility Department of the Jagiellonian University. Tissue samples (average dimensions 0.5 × 0.5 × 0.5 cm) were mixed with Complete proteinase inhibitor cocktail (Roche, Germany) and homogenized on an ice-bath in a glass-glass Potter-Elvejhem homogenizer. The resulting suspensions were mixed with an equal volume of SDS sample lysis buffer (4% SDS, 20% glycerol, 125 mM Tris-HCl pH 6.8), boiled in a water bath for 15 minutes, and spun down. The supernatants were used for further analysis. A detailed description of the tissue preparation and semi-quantitative assessment of RCAS1 and beta-Actin relative amounts in examined tissue samples using Western blot technique was presented in our previous report [16,18].

### Western blotting

The total protein content in the obtained supernatants was measured using a BCA assay kit. Different sample volumes equivalent to 50 μg/4 g of total protein were loaded on SDS-PAGE tris-tricine gels (Shagger & von Jagow, 1987). Pre-stained broad range molecular mass protein standard (BioRad, USA) was used in the marker lane. After electrophoresis gels were electrotransferred on Immobillon-P polyvinylidene difluoride (PVDF) 0.45 μm membrane (Millipore, USA) in a buffer containing 10 mM 3-(cyclo-hexyloamino)-1-propanesulfonic acid (CAPS).

The obtained membrane blots were blocked by overnight agitation in bovine serum albumin in TST buffer (0.01 M Tris-HCl, pH 7.4, 0.9% NaCl, 0.5% Tween-20). The membranes were then agitated in mouse monoclonal anti-RCAS1 MoAb 22-1-1 IgM-class antibody (Medical and Biological Laboratories, Japan) in TST buffer. After discarding the solution and 4 cycles of washing in TST buffer, the membranes were immersed and agitated in biotinylated anti-mouse IgM μ-chain specific antibody in TST buffer and, after 4 cycles of washing, in ExtraVidin-alkaline phosphatase conjugate in TST. After washing, the color of a band was developed using FastRed TR/Naphtol AS-MX tablet set. All steps of the procedure were performed at room temperature. Finally, using Western blot method and monoclonal anti-beta-Actine mouse antibody, the relative quantity of beta-Actine was estimated as a control.

### Computer- aided image analysis

Fluor-S MultiImager (BioRad, USA) was used to scan the membranes and the computer program QuantityOne (BioRad, USA) was used to quantify band intensities. All calculations were performed for the RCAS1 antigen having a molecular mass of about 32 kDa [17]. The intensities of this band were expressed in arbitrary relative units, and one unit (U) was defined as the band intensity produced by the reference sample, randomly chosen, yet the same on all blots, and always applied in the same amount. Because all immunoblots contained the same RCAS1 quantity standard and all lanes were loaded with the same amount of total protein (50 μg/4 g), the values determined were highly repetitive and independent of the conditions of the experiment. The results indicated the relative amount of RCAS1 32 kDa antigen in 50 μg/4 g of total sample protein.

### Immunohistochemistry

Two independent observers with no knowledge of the clinicopathologic data reviewed the immunohistochemical expression of RCAS1. Immunohistochemical analysis was performed in the Pathomorphology Department of the Jagiellonian University. 5 μm thick slides were deparaffinized, rehydrated, and rinsed in distilled water. Endogenous peroxidase activity was blocked by 8 minutes of incubation in 3% H2O2 at room temperature. The slides were then rinsed and immersed in boiling citrate buffer (pH 6.0) in a microwave oven with three changes of buffer for 5 minutes each. For the immunolocalization of RCAS1 the slides were treated with the mouse monoclonal antibody Anti-RCAS1 (Medical and Biological Laboratories, Naka-ku Nagoya, Japan in DAKO Antibody Dilutent with Background Reducing Components-DAKO, Denmark, dilution 1:1000) in a moist chamber overnight. The slides were subsequently rinsed in TBS buffer (pH 7.6) and incubated with secondary antibody (DAKO Envision TM+ System Labelled Polymer HRP – anti-mouse (DAKO, Denmark) for 30 minutes at room temperature. Visualization was performed using AEC (3-amino-9-ethyl-carbazole) as a chromogen (AEC Substrate Chromogen ready-to-use, DAKO, Denmark) for 10 minutes at room temperature. The sections were counterstained with hematoxylin and mounted in glycergel. We assumed that positive reactivity for RCAS1 occurred when we observed a granular cytoplasmic (rarely membranous) staining pattern in at least 10% of cells with the following intensity: 0- no reactivity; 1-weak (10–25% positive cells); 2-moderate (26–50% positive cells); 3-overexpression (51–100% positive cells).

### Statistical analysis

The distribution of subjects was analyzed using the Shapiro-Wilk's test. The resulting data and control data were compared using Student's t-test (the relative amount of RCAS1) and the Mann-Whitney test (β-Actin). Significance was accepted at p < 0.05.

## Results and discussion

### The expression of RCAS1 in laryngeal and pharyngeal squamous cell carcinoma and clear surgical margin

**Figure 1:**
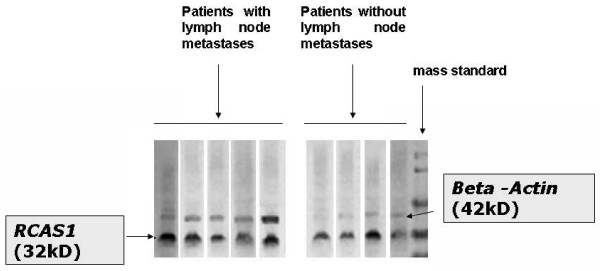
**In Western blot technique, RCAS1 was identified as a 32 kDa band, while beta-actin control protein corresponded to 42 kDa band**. Each column corresponds to a tissue sample derived from a cancer nest.

The Western blot analysis revealed 32 kDa bands corresponding to RCAS1 protein and 42 kDa bands corresponding to beta-Actin, control protein, Figure 1[17,18].

RCAS1 protein was not detected in the tissue samples derived from the healthy mucous membranes of the upper respiratory tract of the patients without cancerous lesions by means of Western blot method.

**Figure 2:**
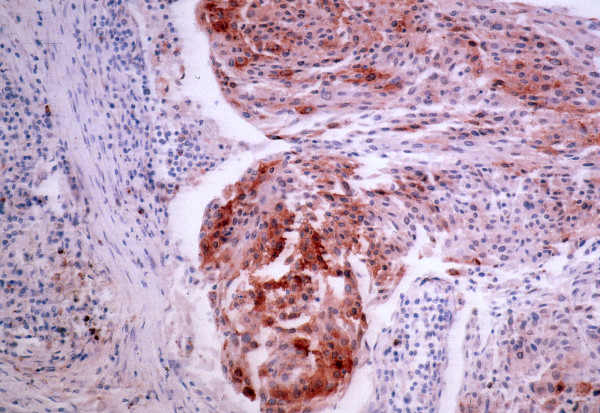
**RCAS1 immunoreactivity was identified as a granular type of staining pattern in cancer specimens**.

**Figure 3:**
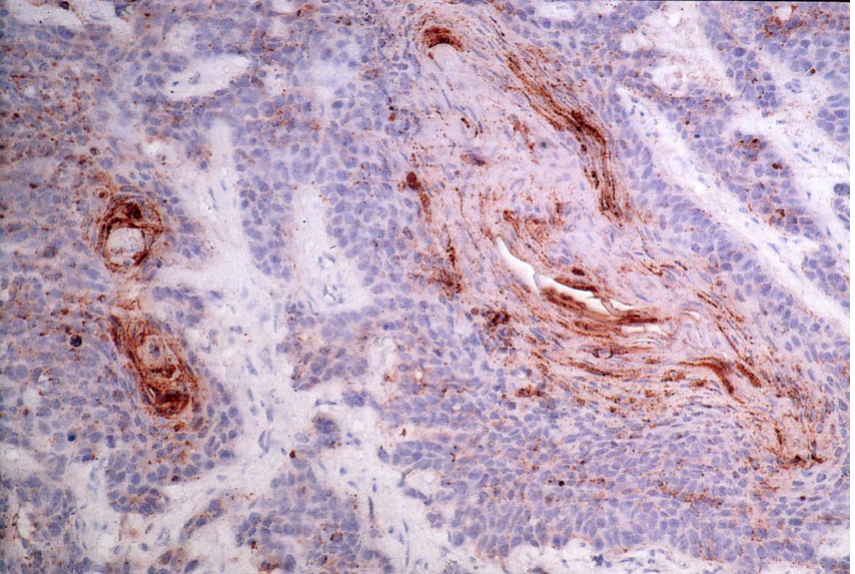
**RCAS1 expression in cancer nest**.

RCAS1 expression was established in all pharyngeal and laryngeal squamous cell carcinoma tissue samples. Immunohistochemical analysis confirmed the expression of RCAS1 in all the cancer tissue specimens. The granular type of diffused cytoplasmic expression was evaluated (Figure 2, Figure 3).

The presence of RCAS1 was also revealed in immunohistochemistry in almost all (96.5%) the samples derived from histopathologically negative surgical margins.

**Figure 4:**
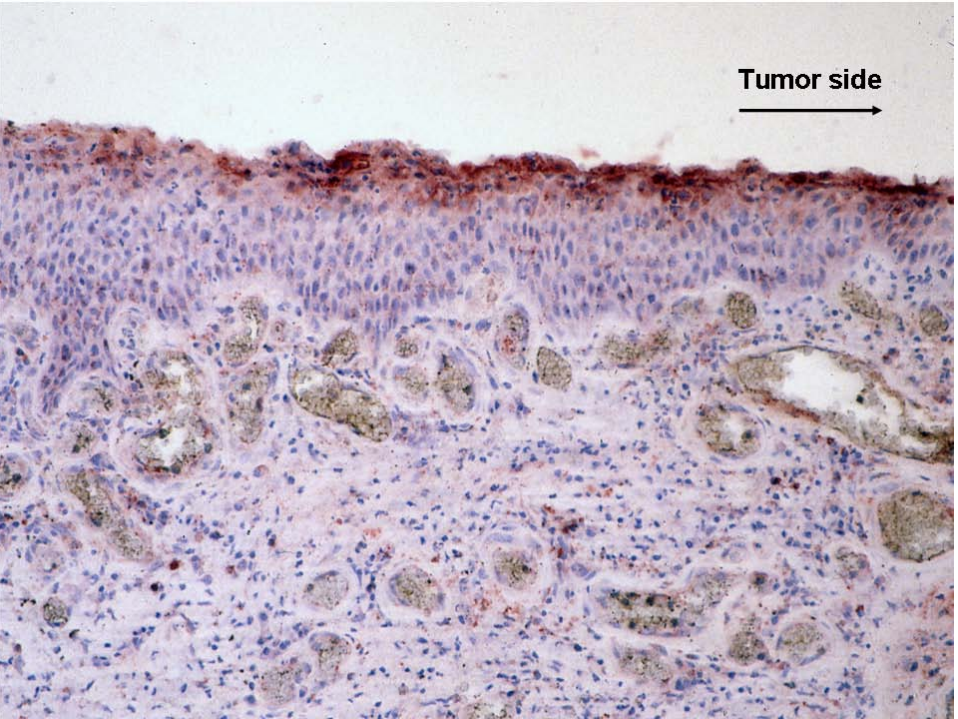
**RCAS1 immunoreactivity in clear surgical margin decreased as the distance from the tumor increased**.

**Figure 5:**
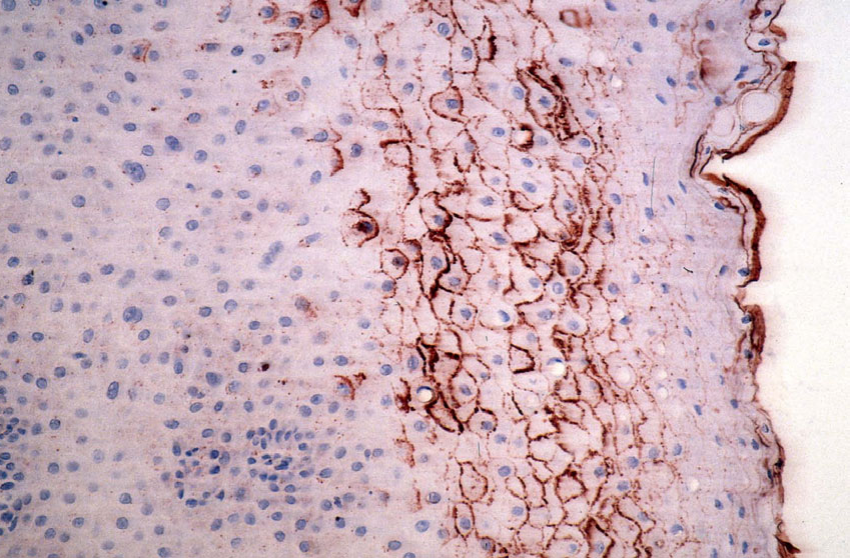
**RCAS1 expression in clear surgical margin presenting membrane staining pattern**.

RCAS1 immunoreactivity in stratified squamous epithelium from healthy surgical margin was present mostly in the superficial layers of the epithelium, while RCAS1 immunoreactivity identified in the stratified columnar epithelium was present only in the margins of the epithelium as a very intense brown reaction. The immunoreactivity of RCAS1 remained at the highest level in close vicinity of the cancer and was observed to decrease as the distance from the tumor increased (Figure 4). The type of staining pattern in the healthy epithelium was diffused granular cytoplasmic and/or membranous (Figure 5).

RCAS1 immunoreactivity was statistically significantly higher in the cancerous tissue specimens than in the clear surgical margin. The results obtained through immunohistochemical analysis remained in compliance with those obtained by the Western blot method. No differences were observed in the average relative amount of β-Actin control protein between examined groups of patients (Table 2, Table 3).

**Table 2:** RCAS1 immunoreactivity in pharyngeal and laryngeal cancer and clear surgical margin derived from the same individuals.

		RCAS1 immunoreactivity			
		
Variables	Number	-	+1	+2	+3
Pharyngeal and laryngeal squamous cell carcinoma	51	0	0	22 (44%)	29 (56%)
Clear surgical margin	51	1 (3.5%)	24 (44.5%)*	25 (48.5%)*	1 (3.5%)

*The change in the intensity of immunoreactivity was observed in six specimens (12%), in one case in the group of the +1 RCAS1 immunoreactivity and in 5 cases in the group of +2 RCAS1 immunoreactivity; the expression decreased with the distance from cancer cells. In four examined samples, the RCAS1 immunoreactivity level in closely tumor-adjacent tissue was established as moderate and decreasing to weak in the line of resection. In two cases, weak RCAS1 immunoreactivity in the directly tumor-adjacent tissue diminished to the point of absence in the resection line.

**Table 3:** The comparison of the average relative amount of RCAS1 as assessed by the Western blot method in carcinoma and cancer-free surgical margin.

	Pharyngeal and laryngeal squamous cell carcinoma (n = 51)	Clear surgical margin (n = 51)	*P*
Average RCAS1 relative amount (± SD)	1.1016 (± 0.6090)	0.891 (± 0.5237)	0.02
Average β-Actin relative amount (± SD)	1.21163 (± 0.648)	1.2286 (± 0.47)	NS
RCAS1/β-Actin ratio	0.909	0.725	-

NS-no statistical significance

SD-standard deviation

We also assessed the RCAS1 expression level with respect to the clinicopathological variables (tumor size, grade, and the presence of lymph node metastases) using Western blot method. No differences were identified in the relative amount of RCAS1 with respect to tumor size. However, the average relative amount of RCAS1 was found to be statistically significantly higher in the samples taken from high-grade cancers (Table 4).

**Table 4:** The comparison of the average relative amount of RCAS1 as assessed by the Western blot method in carcinoma and cancer-free surgical margin in relation to the tumor grade.

	Average relative amount of RCAS1 ± SD		
		
Tumor grade	G2 (n = 31)	G3 (n = 19)	*P*
Pharyngeal and laryngeal squamous cell carcinoma	0.987 (± 0.522)	1.2211 (± 0,6937)	0.04
Clear surgical margin	0.6922 (± 0.4699)	0.996 (± 0.3808)	0.03

SD-standard deviation.

No differences were observed in the average relative amount of β-Actin control protein.

The average relative amount of RCAS1 was identified as significantly higher in patients with lymph node metastases in comparison to patients without lymph node metastases (Table 5).

**Table 5:** The comparison of the average relative amount of RCAS1 as assessed by the Western blot method in laryngeal and pharyngeal cancer specimens with respect to the presence of lymph node metastases.

Lymph nodes	N negative (n = 24)	N positive (n = 27)	*P*
RCAS1 average relative amount in cancer (± SD)	0.984 (± 0.5883)	1.287 (± 0.5504)	0.04
Average β-Actin relative amount in cancer (± SD)	1.132 (± 0.532)	1.282 (± 0.723)	NS
RCAS1/beta Actin ratio in cancer	0.862	1.003	-
RCAS1 average relative amount in clear surgical margin (± SD)	0.805 (± 0.550)	0.869 (± 0.483)	NS
Average β-Actin relative amount in clear surgical margin (± SD)	1.072 (± 0.473)	1.192 (± 0.386)	NS
RCAS1/beta Actin ratio in clear surgical margin	0.75	0.72	-

SD-standard deviation

The average relative amount of RCAS1 was also statistically significantly higher in patients with the presence of lymph node metastases in which the presence of the extracapsular spread of cancer infiltration was observed than in patients who did not exhibit the extracapsular spread (Table 6).

**Table 6:** The comparison of the average relative amount of RCAS1 as assessed by the Western blot in laryngeal and pharyngeal cancer specimens obtained from patients with lymph node metastases with and without the presence of extracapsular spread.

Lymph nodes	The presence of extracapsular spread (n = 16)	No extracapsular spread (n = 11)	*P*
Average relative amount of RCAS1 in cancer (± SD)	1.4631 (± 0.5285)	0.8681 (± 0.4362)	0.0011
Average β-Actin relative amount in cancer (± SD)	1.034 (± 0.731)	1.102 (± 0.481)	NS
RCAS1/beta Actin ratio in cancer	1.414	0.787	-
RCAS1 average relative amount in clear surgical margin (± SD)	0.859 (± 0.529)	0.831 (± 0.500)	NS
Average β-Actin relative amount in clear surgical margin (± SD)	1.1806 (± 0.477)	1.058 (± 0.472)	NS
RCAS1/beta Actin ratio in clear surgical margin	0.727	0.785	-

SD-standard deviation

### RCAS1 expression and the recurrence of the disease

We assessed the expression of RCAS1 in cancer and in clear surgical margin derived from all examined patients with respect to the recurrence of the cancer process by evaluating a group of 19 patients who had suffered a relapse during the period of observation following surgical treatment.

Western blot analysis revealed that the level of RCAS1 expression in clear surgical margin was statistically significantly higher in tissues derived from patients who had experienced a recurrence of the disease than in patients who had recovered. Interestingly, no differences between the two groups of patients with respect to RCAS1 expression levels in the cancer cells themselves were found using both biochemical techniques. Additionally, no differences were observed between the examined groups with respect to beta-Actin control protein (Table 7).

**Table 7:** The comparison of the average relative amount of RCAS1 in laryngeal and pharyngeal squamous cell carcinoma and in its clear surgical margin with respect to disease recurrence within one-year follow up.

	No Recurrence (n = 32)	Recurrence (n = 19)	*P*
RCAS1 average relative amount in pharyngeal and laryngeal squamous cell carcinoma (± SD)	1.097 (± 0.5505)	1.1067 (± 0.6874)	NS
RCAS1 average relative amount in clear surgical margin (± SD)	0.5504 (± 0.3086)	1.2575 (± 0.4579)	0.0002

A strong immunoreactivity level in the clear surgical margin was observed in one patient who had experienced a recurrence of the disease 6 months after radical surgical treatment. Cancer recurrence was also observed in 78% of patients with a moderate level RCAS1 immunoreactivity within the clear surgical margin. In the group with a weak level of RCAS1 reactivity in the clear surgical margin, only 15% of the patients experienced a recurrence. Moreover, the RCAS1 immunoreactivity level was observed to decrease as the distance from the tumor increased. In cases of cancer recurrence, the RCAS1 level ranged from moderate in the tissues adjacent to the tumor to weak in the resection line. There were no recurrences in patients in whom the RCAS1 immunoreactivity level ranged from weak in the tissue adjacent to the tumour to absent in the resection line (Table 8).

**Table 8:** RCAS1 immunoreactivity in pharyngeal and laryngeal cancer and its clear surgical margin in the patients who developed a recurrence of the disease within the observation period following surgical treatment.

		RCAS1 immunoreactivity			
		
Variables	Number	-	+1	+2	+3
Pharyngeal and laryngeal squamous cell carcinoma	19	0	0	11 (57%)	8 (43%)
Clear surgical margin	19	0	3 (14,6%)	15 (78%)	1 (7,4%)

RCAS1 expression was confirmed by Western blot and immunohistochemical methods in all pharyngeal and laryngeal squamous cell carcinoma specimens and in almost all histopathologically clear margins, but was not detected in the healthy mucous membranes derived from the upper respiratory tract of the patients without cancerous lesions. RCAS1 expression has been reported in various cancers and its expression has been associated with such markers of poor prognosis as tumor size, the presence of lymph node metastases, a high grade of tumor cells, and the depth of tumor invasion [8-18]. RCAS1 expression has been detected in uterine cervical cancer by Sonoda *et al.*, and it has been proposed that this expression is related to tissue remodeling. Sonoda *et al. *has concluded that RCAS1 expression might be responsible for the depth of tumor invasion [6,19]. Moreover, the study determined that RCAS1 was significantly associated with the expression of VEGF and microvessel density. Consequently, it was concluded that RCAS1 might be responsible for regulating the angiogenesis of the tumor and in this way might control tumor growth [19].

As in these reports, we found that RCAS1 expression was associated with high tumor grade and the presence of lymph node metastases, especially with extracapsular spread. RCAS1 expression in pharyngeal and laryngeal healthy surgical margin would thus seem to be very significant. Initially, the RCAS1 staining pattern in all the specimens – both the cancer specimens and their clear surgical margins – appeared to be the same diffused granular staining pattern. However, the pattern was always present in the cytoplasm but was rarely seen on cell membranes. Furthermore, the RCAS1 content was high in the cancerous cells and its expression decreased as the distance from the tumor increased (Figure 4).

To our knowledge, this is the first investigation to focus on RCAS1 stroma expression in pharyngeal and laryngeal cancer with respect to cancer relapse.

In the present study, a high level of RCAS1 expression in healthy surgical margin, including healthy stroma, was associated with a higher frequency of local cancer recurrence within the period of observation. The participation of the stroma in cancer invasion is an intriguing finding. In our previous report, MT expression was identified in the healthy tissue adjacent to cancerous tumors of the head and neck and breast [22]. The tumor environment has been typified by the MT over-expression that may result from the destruction of local physiological processes regulating the stromal invasion [22].

The biological role of RCAS1 involves the inhibition of activated lymphocytes and the induction of the apoptosis of activated CTLs and NK cells [8,17,20]. The level of RCAS1 expression has been found to be inversely related to the infiltration of CD3 positive lymphocytes of a tumor in breast cancer [23,24]. Moreover, RCAS1 expression has been associated with the number of apoptotic lymphocytes adjacent to tumor cells in both lung cancer and Hodgkin's disease [9,24]. An increase in the apoptosis of lymphocytes (mainly CD3+) has also been observed in uterine cervical cancer surrounding RCAS1 positive-cancer cells and RCAS1-positive metastatic cancer cells in lymph nodes [6].

Finally, we have recently described the participation of RCAS1 in the regulation of cytotoxic immune response in: Waldeyer's ring tissue, nasal polyps, the endometrium, and the placenta [25-29].

RCAS1 would therefore seem to play an important role in regulating immune responses. Its expression in the placenta during pregnancy enables the development of the maternal immune tolerance. Furthermore, RCAS1 decreases before labor, making the maternal immune tolerance phenomenon reversible [29,30]. RCAS1 has also been found in the epithelium of nasal polyps, as well as in their microenvironment, where its expression by macrophages (CD68 positive cells) has been confirmed. It is thus involved in the regulation of the local chronic inflammatory process [25]. Cancer is characterized by two phenomena related to RCAS1 expression: one is the chronic perpetual inflammation accompanying cancer; the other phenomenon is that of the host immune tolerance which enables tumor growth. In both situations, RCAS1 may participate in the chronic inflammatory process as an important suppressor of the anti-tumor immune response as well as in the development of pro-tumor tolerance. RCAS1 expression in the tumor has been shown to increase tumor invasiveness [7-19], but its expression by the healthy epithelium of adjacent tissues may result from the increase in local immune deregulation, making the tolerance irreversible and facilitating tumor invasion in tumor-tolerant adjacent tissues. This may be why we observed a recurrence of the disease in the patients with high levels of RCAS1 expression in the clear surgical margins while patients who exhibited no RCAS1 expression in the tumor margins or in the resection line had no relapse.

Like Sonoda *et al. *in the study [7] on endometrial cancer, we have demonstrated that in pharyngeal and laryngeal cancer patients, the sRCAS1 blood serum concentration level increases as the tumor progresses. Moreover, as was also observed in the *Sonoda et al. *study [7], sRCAS1 concentration level in head and neck cancer patients has been found to decrease following surgical treatment but to increase again if the cancer recurs [30,7]. Furthermore, the blood serum sRCAS1 concentration level in squamous cell carcinomas has been shown to be higher in the secondary cancer than it was in the primary cancer (before the surgery). A tumor suppresses immune system activity not only locally, but also generally, and the effect of this suppression is long term [31]. Most likely the increase of sRCAS1 blood serum concentration associated with cancer relapse indicates that sRCAS1 might suppress the immune system generally, thus enabling the relapse. The expression of RCAS1 in clear surgical margin also seems to be related to the local suppression of cytotoxic immune cells around the tumor that along with tissue remodeling may facilitate tumor invasion, thus resulting in cancer relapse.

## Conclusion

In conclusion, the selective cytotoxic immune cell suppression concomitant with tumor growth and connected with locally occurring RCAS1 expression (stromal RCAS1 expression) seems to be an important event related to cancer recurrence.

## Competing interests

The authors declare that they have no competing interests.

## Authors' contributions

MDW conceived of the study, participated in the sequence alignment and the study design, was responsible for the analysis of the data and the manuscript creation. RT carried out the histopathological and immunohistochemical analyses of the clinical material. AL carried out the histopathological and immunohistochemical analyses of the clinical material. LW carried out the Western blot analysis, performed the statistical analysis, cooperated in the preparation of the manuscript. JS participated in the preparation of the manuscript. All authors read and approved the final manuscript.

## Pre-publication history

The pre-publication history for this paper can be accessed here:

http://www.biomedcentral.com/1471-2407/9/35/prepub
